# Progress of Surgery

**Published:** 1901-03-23

**Authors:** 


### Progress of Surgery.

(*Continued from page* 403.)

### GENITO-URINARY AND RECTAL—(*continued*).

**Stricture of the Rectum.**—F. C. Wallis[6] has contributed a paper on the causes and treatment of the non-malignant forms of this affection. Such strictures, he points out, are invariably the sequel of pre-existing ulceration. The causes of this ulceration are said to be syphilis, tubercle, and dysentery. Obstetric trauma and rectal operations are spoken of vaguely as occasional causes of stricture. Most writers regard syphilis to be the cause of a large percentage of rectal strictures. Wallis believes that all cases of ulceration and stricture of the rectum which could not be accounted for in any other way have been put down to syphilis without any real evidence that the cause was syphilis, and that syphilis really accounts for comparatively few of the cases. The most careful questioning usually fails to elicit any evidence of that disease. The majority of the cases are in women of about the age of 30, an age period in which gummata are not common, and the usual anti-syphilitic remedies are useless. After the war of the rebellion in America, not a single case of stricture attributable to dysentery occurred. Tuberculous ulceration is considered quite a rare cause. Wallis believes the most common cause of rectal stricture is septic ulceration. This may be started by septic discharge from the outside, attacking an abraded portion of the mucous membrane, or result from post-operative sepsis, or from protracted labour leading to necrosis of an area of rectal mucous membrane. Ulceration once started the continual contact of fæces renders it progressive. Wallis then notices a clinical feature in cases of rectal ulceration which is of interest, and has apparently not been before observed, viz. that attacks of acute synovitis are apt to occur. Three cases illustrating this are quoted. The complication is no doubt of septic origin. Two cases illustrating the production of rectal ulceration from sepsis following operations are given. The methods of treatment of stricture of the rectum are then criticised. The passage of bougies, Wallis considers, tends to keep up ulceration. The great trouble due to the fact that natural peristalsis and muscular movements of the bowel end at the stricture cannot be affected by bougies, and posterior proctotomy does not affect it. In the latter case the stricture recurs as badly as ever, and possibly becomes worse than before, when the wound granulates up. These and other considerations have led Wallis in some cases to excise the strictured portion of the bowel, bringing down the healthy gut from above to the anal margin. A preliminary colotomy is desirable. The hæmorrhage in the operation is often severe. The peritoneum may have to be freely opened. Post-operative shock may be alarming. The notes of two cases in which extensive operations of this kind were performed are given in the paper. The results were very satisfactory.

**Prolapse of the Rectum.**—Gérard Marchant[7] supports the view that prolapse of the rectum is due to yielding of the rectal wall at the level of the vesico-rectal *cul-de-sac*, the yielding being caused by the pressure of the small intestine lodged in the pouch of Douglas, and by constipation weakening the rectal walls. The anterior wall of the rectum is first protruded, and as it descends it carries with it the lateral and posterior walls, and thus a complete prolapse results. It is held that if this view of the origin of prolapse is correct, surgical treatment should be directed with the objects of diminishing the length and width of the protruded portion of the rectum and of suspending this to some unyielding structure. A case successfully operated upon by Pauchet after the manner devised by Marchant is referred to. The operation consists in (1) contracting the anus by partially excising it; (2) folding the walls of the prolapsed part by sutures passed vertically and from side to side to diminish its size; and (3) fixing the contracted intestine to the posterior pelvic wall (rectococcypexis or colopexis).

**Wounds of the Rectum.**—Thomas H. Manley,[8] of New York, has written on this subject. He points out that in a healthy subject superficial wounds in the ano-rectal outlet heal with equal facility as those situated in more salubrious situations. He mentions a boy of nine years of age whom he treated for deep lacerations of the anus, the results of pæderasty—the rents extending one backwards and one forwards. Another case was that of a heavy man who, when intoxicated, sat down on a decanter. He walked to hospital, and when he entered was in great distress, and blood was flowing down into his boots. The serrated broken edge of the glass was felt an inch above the anus. The stopper came away three days later, after an enema. Many accidents have resulted from the use of rectal instruments, especially injection apparatuses, bougies, and sounds. Manley operated on a case in which nearly a gallon of oil and soapsuds had been forced through the sigmoid flexure by the long rectal tube. Instruments may produce contusions which perforate later. The use of sounds in rectal strictures has several times caused perforation, and cases are mentioned. Cases have resulted from attacks by bulls. Cases in which the bladder has been perforated and yet which made good recoveries are alluded to. Penetrating wounds injuring the omentum, the jejunum and liver, the psoas muscles and the diaphragm are mentioned. Researches have shown that the mucous membrane offers the least resistance to the contusive force. Otis recorded 103 cases of gunshot wound of the rectum. Treatment is briefly considered. Otto G. Ramsay,[9] of the John Hopkins University, has a paper on a case in which a pin was found embedded in the rectum. The patient, a woman of 55, had been seized with sharp pain in the rectum and constant rectal tenesmus two weeks before. The pain was stabbing, and very severe when she exercised or when the bowels were moved. The pin was felt on rectal examination to lie across the lumen of the bowel with its head buried and the point free and impinging on the opposite wall. There was no sign of abscess formation. He easily removed the pin. The modes in which the pin could have reached this position and been embedded in such a way are discussed. Most likely it had been swallowed. Pins and other bodies swallowed have often been found in the vermiform appendix. Such pins have evidently been swallowed. Similarly, fish bones have been found in the rectum. In this case probably the head of the pin became engaged in one of the rectal pouches, and then caused ulceration and became embedded. The passage of fœces apparently caused the pin to lie across the lumen of the bowel, and then the symptoms began. If the point had penetrated the bowel, probably it would have gone deeper and an abscess would have resulted. The site of puncture from such bodies is usually within the last inch of the rectum, as shown by Goodsall, who also states that the time the object takes to pass from the month is from one to nine days. An abscess or fistula is the most frequent sequence. A foreign body has often caused multiple recurrences of abscesses, and should be looked for in cases of rectal and perirectal abscess.

[6] Brit. Med. Jour., Oct. 1900. [7] *Vide* Brit. Med. Jour. (Epitome), Nov. 1900. [8] Med. Brief, Aug. 1900. [9] John Hopkin's Hosp. Bulletin, Oct. 1900.

